# Challenges in measuring angles between craniofacial structures

**DOI:** 10.1590/1678-7757-2018-0380

**Published:** 2019-06-03

**Authors:** Marilia Yatabe, Liliane Gomes, Antonio Carlos Ruellas, Julia Lopinto, Lucie Macron, Beatriz Paniagua, Francois Budin, Juan Carlos Prieto, Marcos Ioshida, Lucia Cevidanes

**Affiliations:** 1 University of Michigan, School of Dentistry, Department of Orthodontics and Pediatric Dentistry, Ann Arbor, Michigan, USA.; 2 Universidade Estadual Paulista (UNESP), Faculade de Odontologia de Araraquara, São Paulo, Brazil.; 3 GE Healthcare, Limonest, France.; 4 Kitware SAS, Villeurbanne, France.; 5 Kitware Inc., Carrboro, North Carolina, USA.; 6 University of North Carolina, School of Medicine, Department of Psychiatry, Chapel Hill, North Carolina, USA.

**Keywords:** Reproducibility of results, Three-dimensional imaging, Computer-assisted image processing, Cone-beam computed tomography

## Abstract

**Objective:**

Three-dimensional (3D) angular measurements between craniofacial planes pose challenges to quantify maxillary and mandibular skeletal discrepancies in surgical treatment planning. This study aims to compare the reproducibility and reliability of two modules to measure angles between planes or lines in 3D virtual surface models.

**Methodology:**

Twenty oriented 3D virtual surface models de-identified and constructed from CBCT scans were randomly selected. Three observers placed landmarks and oriented planes to determine angular measurements of pitch, roll and yaw using (1) 3D pre-existing planes, (2) 3D planes created from landmarks and (3) lines created from landmarks. Inter- and intra-observer reproducibility and repeatability were examined using the Intra-Class Correlation (ICC) test. One observer repeated the measurements with an interval of 15 days. ANOVA was applied to compare the 3 methods.

**Results:**

The three methods tested provided statistically similar, reproducible and reliable angular measurements of the facial structures. A strong ICC varying from 0.92 to 1.00 was found for the intra-observer agreement. The inter-observer ICC varied from 0.84 to 1.00.

**Conclusion:**

Measurements of 3D angles between facial planes in a common coordinate system are reproducible and repeatable either using 3D pre-existing planes, created based on landmarks or angles between lines created from landmarks.

## Introduction

Quantification of facial characteristics is of extreme importance in diagnosis and different measurement techniques have evolved from direct measurements of skulls to indirect measurements based on imaging exams. The advent of radiographs markedly increased the number of studies that attempted to understand the development of growth and treatment results.^1^ ^–^ ^3^ Even though 2D images had provided important information for decades, nowadays 3D images are able to provide more accurate information, therefore improving the assessment of craniofacial anatomy and changes after treatment or surgery. It overcomes some inherent flaws of 2D images, such as patient's head position, superimposition of different anatomical structures, image magnification and distortions. It also enables volumetric measurements, allowing for a detailed assessment of maxillofacial structures in variable thickness of axial, coronal and sagittal slices, providing real measurements with no magnification.^4^ ^–^ ^13^

Cone-beam computed tomography (CBCT) has been the image of choice for diagnosis and treatment planning of reconstructive surgeries, dental implants, patients with asymmetry and/or craniofacial anomalies because it improves the visualization and understanding of the anatomy. However, caution should be taken when requesting these images due to its radiation dose. 3D cephalometric tools for clinical diagnosis as well as visualization of the 3D images have been available in numerous commercial software such as Mimics (Materialise, Leuven, Belgium), OsiriX (Pixmeo, Geneva, Switzerland), Dolphin3D (Dolphin Imaging & Management Solutions, Chatsworth, California, USA), InVivo Dental (Anatomage, San Jose, California, USA), and Ondemand3D (CyberMed, Seoul, Korea). The visualization tools allow for the assessment of CBCT images by not only showing axial, coronal and sagittal images, but also creating a 3D reformatted image. However, research purposes and surgery planning go beyond simple visualization, and therefore several 3D cephalometric tools have also been proposed to quantify linear and angular craniofacial measurements, transitioning from 2D to 3D analyses.^14^ ^–^ ^20^ Most of these studies use CBCT to visualize a specific region, but still perform an overall overview of the patient using reformatted 2D images.

The use of 3D planes to quantify the craniofacial morphology proportions or measure angles between planes of anatomical structures pose mathematical challenges. It is important to understand that in 3D analysis a plane is defined by three points that may not lie at the same level; that the angle between two planes are determined by the normal vector of the planes; and clinicians need to become familiar with which angle to measure in the 3D space, since complementary angles are calculated. The main purpose is to present three new methods from an open-source software (SlicerCMF) to calculate anatomical angles in virtual surface models (constructed from CBCT images) in the three planes of space and assess the reproducibility of measuring the angles: (1) using 3D pre-existing planes, (2) creating a 3D plane based on landmarks, and (3) using lines created from landmarks.

## Methodology

This study was approved by the Institutional Review Board (HUM00066254). Sample size calculation was performed by three observers a minimum and expected ICC (Intra-Class Correlation) of 0.75 and 0.90, respectively. As a result, a sample size of 19 models was needed for inter-observer correlation, and 30 models for intra-observer correlation.

Three-dimensional surface models of de-identified patients were randomly selected from the archives of the Orthodontic Imaging Lab from the University of Michigan. The only exclusion criterion was lack of well-defined anatomical structures in the 3D surface models. All models were pre-oriented using the midsagittal plane, the Frankfurt horizontal plane, and the transporionic line.^21^

**Figure 1:**
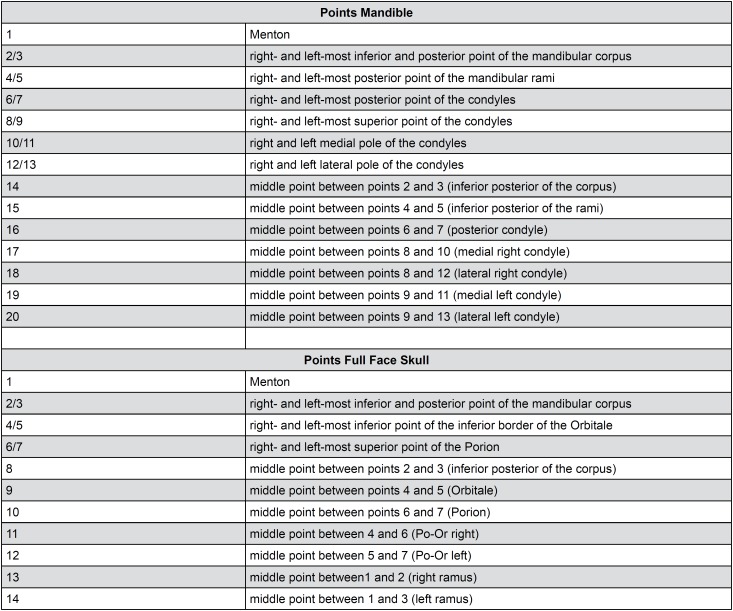
Anatomical landmarks used to calculate angles

**Figure 2:**
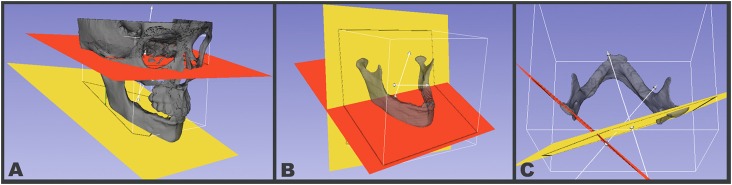
(A) Lateral view of the mandibular (yellow, inferior position) and Frankfurt (red, superior position) planes used to calculate FMA roll and pitch in the coronal and sagittal views, respectively; (B) Posterior border of the ramus plane (yellow, vertical) and Mandibular plane (red, inferiorly located) used to calculate the Gonial Angle pitch in the sagittal view. Note indication of main (α) and complementary (ρ) angles measured; (C) Left Condyle (red; left) and Right Condyle (yellow; right) planes used to calculate the Condylar Angle yaw in axial view

Utilizing three angular measurements commonly used in 2D cephalometry analysis, in either lateral, A-P or submentovertex cephalograms, a list of clearly defined anatomical landmarks that were placed in the surface models can be found in Figure 1. Anatomical angles were then measured in different views of the 3D space, defined as follows:

1. FMA (Frankfurt-mandibular plane): The Frankfurt Horizontal plane passes by the most superior portion of the “Porion” at both sides and by the most inferior portion of the “Orbitale” at both sides. In cases of asymmetry, the plane was positioned in-between the most inferior portion of the left and right sides. When placing the landmarks, the software is able to create a mid-point between the right- and left-most inferior portion of the Orbitale. The Mandibular plane passes by the most inferior and posterior portion of the lower border of the mandibular corpus (right and left sides) and by the “Menton” point (Mandibular Plane). This angle was assessed in coronal (FMA roll) and sagittal (FMA pitch) views (Figure 2a).
2. Gonial Angle: The “Mandibular plane” passes by the most inferior and posterior portion of the right and left lower border of the mandibular corpus and by the “Menton” point. The “Posterior border of the ramus plane” passes by the most posterior points of right and left condyles and rami. In cases of asymmetry, the plane was positioned in between the most posterior portion of the left and right condyles. When placing the landmarks, the software is able to create a mid-point between the right- and left-most posterior portion of the Condyles. This angle was assessed in sagittal view (Figure 2b).
3. Condylar Angle: Each condylar long axis plane passes through the medial and lateral poles, and through the center of the superior surface of the condylar head at each side. The condylar angle was assessed in axial view (Figure 2c).

**Figure 3:**
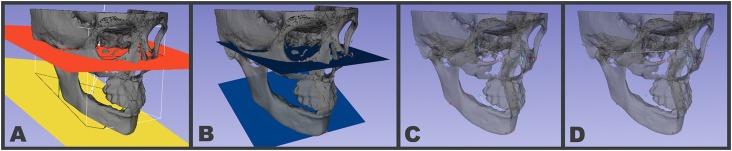
(A) The original coordinate planes in the SlicerCMF 3.1 (www.slicer.org) software were manually moved to intersect the anatomical structures; (B) Illustrating the blue planes created by placing landmarks in the model; (C) Illustrating anatomical landmarks placed over the 3D surface model. Lines created to represent the planes to measure pitch and (D) roll

The intersection of lines and/or planes in 3D determines the Yaw, Pitch and Roll angles in the three spatial planes (axial, sagittal and coronal, respectively). Three different methods for calculating the anatomic angles between planes were tested:

- 3D pre-existing planes: Using the Angle Planes module in SlicerCMF 3.1 (www.slicer.org), two pre-existing planes (Axial, Coronal or Sagittal) were manually positioned tangent to each anatomic structure of interest in the 3D surface models in order to determine the angles according to different spatial views (Figure 3a).
- Creating a 3D plane based on landmarks: Using the Angle Planes module in 3D SlicerCMF 3.1 (www.slicer.org), landmarks were placed at specific anatomical locations (Figure 1) in order to create each of the planes used to determine the angles of interest (Figure 3b).
- Angle between lines from landmarks: Using the Q3DC module in SlicerCMF 3.1 (www.slicer.org), landmarks were placed at specific anatomical locations (Figure 1), in order to create lines for the representation of the planes. When assessing bilateral structures, a mid-point was used as representative of both sides (Figure 3c and 3d).

### Statistical analysis

To assess the reproducibility and reliability of the methods, three observers assessed the angles defined in the three spatial planes. To assess the repeatability of the method, one observer repeated the angular measurements with an interval of 15 days. Statistical analysis was performed using the Intraclass Correlation Coefficient (ICC) test.

To compare the three methods of measuring 3D angles while considering the normal distribution of the results, the ANOVA test was applied.

## Results

Table 1 shows the intra- and inter-rater correlations. For the measurements performed with 3D pre-existing planes adjusting the tangent to surfaces by utilizing the Angle Planes module, the smallest intra-observer ICC was 0.93 and the smallest inter-rater ICC was 0.84, For the measurements with “angles between lines from landmarks”, utilizing the Q3DC module, the lowest intra-observer ICC was 0.92 and the lowest inter-rater ICC was 0.88. For the measurements performed with 3D planes created based on landmarks by utilizing the Angle Planes module landmarks option, the lowest intra-observer ICC was 0.94 and the lowest inter-rater ICC was 0.91.

**Table 1:** Intra- and inter-observer correlation assessment

		Q3DC			Angle Planes using Landmarks			Angle Planes using Planes		
		Obs1 x Obs2	Obs1 x Obs3	Obs2 x Obs3	Obs1 x Obs2	Obs1 x Obs3	Obs2 x Obs3	Obs1 x Obs2	Obs1 x Obs3	Obs2 x Obs3
Intra-Observer (Obs1)	Gonial Angle	1			1			0.99		
	Condylar Angle	0.96			0.94			0.97		
	FMA Pitch	1			1			0.99		
	FMA Roll	0.92			0.95			0.93		
Inter-Observer	Gonial Angle	1	1	1	1	1	1	1	0.96	0.97
	Condylar Angle	0.98	0.95	0.95	0.91	0.93	0.91	0.91	0.87	0.84
	FMA Pitch	1	0.99	0.99	1	0.99	0.98	0.98	0.97	0.91
	FMA Roll	0.94	0.97	0.88	0.97	0.98	0.95	0.93	0.93	0.97

Even though there were slight differences in the inter and intra-rater correlations using the three methods for angular measurements, the ANOVA test showed no significant difference between the three methods (Table 2).

**Table 2:** Mean, standard deviation and ANOVA results comparing the 3 methods for each observer

Variables	Observer	Q3DC	Angle Planes - Landmarks	Angle Planes - Planes	ANOVA p value
Gonial angle	Obs1	128.62 (8.96)	128.71 (8.81)	127.34 (7.94)	0.853
	Obs2	128.74 (8.77)	128.59 (8.75)	129.23 (8.79)	0.971
	Obs3	128.75 (9.05)	128.55 (8.91)	128.44 (8.81)	0.994
Condylar Angle	Obs1	133.54 (14.78)	135.24 (15.19)	133.74 (15.50)	0.928
	Obs2	132.78 (15.14)	135.22 (13.88)	134.11 (13.81)	0.864
	Obs3	131.33 (14.37)	134.07 (12.79)	131.65 (14.26)	0.791
FMA pitch	Obs1	28.50 (8.18)	28.70 (8.18)	28.77 (7.84)	0.994
	Obs2	28.41 (8.24)	28.49 (8.18)	28.70 (8.29)	0.993
	Obs3	28.01 (8.17)	28.07 (8.20)	28.47 (8.25)	0.982
FMA roll	Obs1	1.28 (1.50)	1.60 (1.59)	1.72 (1.46)	0.64
	Obs2	1.20 (1.49)	1.81 (1.71)	1.89 (1.69)	0.35
	Obs3	1.28 (1.59)	1.68 (1.58)	1.81 (1.54)	0.527

## Discussion

In the transition between 2D and 3D assessments of craniofacial structures, 2D images have been rendered from CBCT scans and conventional cephalometric analysis has been applied in a number of recent studies. The advantages of cephalometric analysis in images rendered from CBCT scans is the lack of magnification of the image and, in asymmetrical cases, the possibility to measure the right and the left sides separately. Previous studies have shown that 2D digital visual treatment objectives are similar to conventional assessment for the maxilla, but less for the mandible due to surgical mandibular changes being more complex than maxillary changes.^4^ Orthognathic surgery planning has improved significantly with the advent of 3D image analysis. Using 3D virtual surface models instead of 2D rendered images has the advantage of planned and customized osteotomies by creating surgical resection guides,^6^ moving the models as needed to place the landmarks in the most accurate position possible, and to use planes to measure different angles in all three views, which helps improve diagnosis and treatment planning.

With the development of 3D cephalometric analysis, commercial software initially offered capabilities of measuring 3D angles between landmarks.^10^ ^–^ ^13^ ^,^ ^22^ A limitation of the method that requires “placing the landmarks” is that even though these are digital landmarks, they are still placed manually, and therefore, are still open to human errors.^5^ A difference of ±2 mm between landmarks placement is acceptable in orthognathic planning without significant impact on clinical decision-making.^8^ Our findings corroborate the literature, showing differences of approximately 2 mm between first and second measurements, as well as between observers (Table 1). However, those repeated differences occurred both when placing landmarks and when moving the planes towards that direction.

Most conventional angular measurements in 2D cephalometry analysis are performed between two lines that often represent 3D planes such as the Frankfurt Horizontal and Mandibular Plane. When measuring the angles formed by the planes or lines in any of the three methods tested in this study, two complementary angles can be formed between two planes or lines, always resulting in 180°. While current cephalometric analyses do not list a “complimentary” angle and only list the specific angles of interest for clinicians, the current version of the software described in this study delineates preset cephalometric analyses and rather gives the user the flexibility to determine whatever measurements they would like to implement. The new tools require clinicians to interpret and understand what they intend to measure in each of the three spatial views (sagittal, frontal and axial). Interestingly, the given angles between two planes may have clinical meaning in two different views of the 3D space. For example, for FMA, both frontal perspective measurements (FMA roll) and sagittal perspective (FMA pitch) evaluate different and helpful aspects of facial morphology.

This study focused on four angles previously frequently utilized in 2D cephalometry studies with lateral cephalograms (FMA pitch and Gonial angle), frontal cephalograms (FMA roll) and submentovertex x-rays (condylar angle). Even though this study utilized measurements derived from known 2D cephalometry populational norms and standards, the 3D surface models constructed from CBCT images allow users to measure any other angles that may be helpful to evaluate complex skeletal discrepancies that were not previously possible to measure in 2D images.^6^ ^,^ ^11^ ^–^ ^13^ The two methods using landmarks were similar, and no significant challenges were noticed. The method of managing pre-existing planes, however, demanded more practice adjusting the planes towards the correct position during the calibration period. ANOVA results showed statistical similarity between the methods (Table 2). The high intra-observer correlation found suggests that all methods are repeatable options for angular measurements of 3D surface models (Table 1). The high inter-observer correlations suggest that all methods are also reproducible (Table 1). Therefore, users may use any tool they feel more comfortable with.

**Figure 4:**
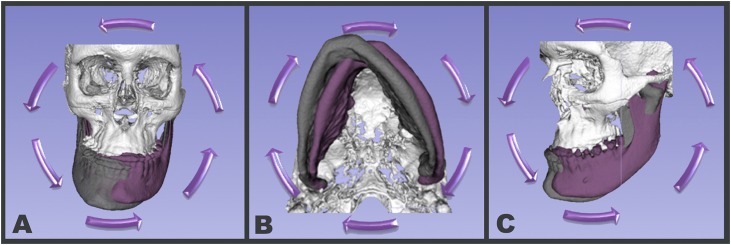
Schematic illustration of roll, yaw and pitch

The greatest challenge in transitioning from 2D to 3D craniofacial measurements is how to interpret the data findings in a clinically meaningful way in order to provide improved diagnosis and assessment of treatment outcomes. While single angular measurements were performed and easily interpreted in a 2D projection of the skull, when 3D angles are measured, three different angles can be determined: pitch, roll and yaw (Figure 4); and it is up to the observer to identify which angles are relevant. Additionally, when comparing between different time-points and different patients, it is important to standardize the head position to consistently assess angular relationships to craniofacial structures. Knowing that 3D measurements are reliable and reproducible, further research should compare them against conventional 2D cephalometry, currently still considered the gold-standard method in research and clinical practice.

## Conclusion

Based on the results from this study, measurements of 3D angles in a common coordinate system are reproducible and repeatable either using 3D pre-existing planes, or creating a 3D plane based on landmarks or angles between lines created from landmarks.
